# Aquaporins Are Essential to Maintain Motility and Membrane Lipid Architecture During Mammalian Sperm Capacitation

**DOI:** 10.3389/fcell.2021.656438

**Published:** 2021-09-01

**Authors:** Ariadna Delgado-Bermúdez, Sandra Recuero, Marc Llavanera, Yentel Mateo-Otero, Andra Sandu, Isabel Barranco, Jordi Ribas-Maynou, Marc Yeste

**Affiliations:** ^1^Biotechnology of Animal and Human Reproduction (TechnoSperm), Institute of Food and Agricultural Technology, University of Girona, Girona, Spain; ^2^Unit of Cell Biology, Department of Biology, Faculty of Sciences, University of Girona, Girona, Spain

**Keywords:** aquaporins, spermatozoa, silver sulfadiazine, cooper sulfate, capacitation, mercury chloride

## Abstract

Aquaporins are a family of ubiquitous transmembrane proteins that allow the transport of water and small molecules across the cell plasma membrane. The different members of this family present a characteristic distribution across different cell types, which is species-specific. In mammalian sperm, different AQPs, including AQP3, AQP7, and AQP11, have been identified; their main roles are related to osmoadaptation and sperm motility activation after ejaculation. Capacitation, which is a post-ejaculatory process that sperm must undergo to achieve fertilizing ability, is triggered by pH changes and different extracellular ions that are present in the female reproductive tract. Considering the function of AQPs and their influence on pH through the regulation of water flow, this study aimed to elucidate the potential role of different AQPs during *in vitro* sperm capacitation using three different transition metal compounds as AQP inhibitors. Cooper sulfate, a specific inhibitor of AQP3, caused a drastic increase in peroxide intracellular levels compared to the control. Mercury chloride, an unspecific inhibitor of all AQPs except AQP7 produced an increase in membrane lipid disorder and led to a decrease in sperm motility and kinetics parameters. Finally, the addition of silver sulfadiazine, an unspecific inhibitor of all AQPs, generated the same effects than mercury chloride, decreased the intracellular pH and altered tyrosine phosphorylation levels after the induction of the acrosome reaction. In the light of the aforementioned, (a) the permeability of AQP3 to peroxides does not seem to be crucial for sperm capacitation and acrosome reaction; (b) AQPs have a key role in preserving sperm motility during that process; and (c) AQPs as a whole seem to contribute to the maintenance of lipid membrane architecture during capacitation and may be related to the intracellular signaling pathways involved in the acrosome reaction. Hence, further research aimed to elucidate the mechanisms underlying the involvement of AQPs in mammalian sperm capacitation and acrosome reaction is warranted.

## Introduction

Cell homeostasis relies on both the integrity and permeability of plasma membrane to water and electrolytes. Because of its amphipathic nature, additional mechanisms to simple diffusion are required in order for water and solutes to pass through the cell membrane (Watson, 2015). Aquaporins (AQPs) are ubiquitous transmembrane water proteins that allow the transport of water and small solutes across cell membranes (reviewed by Yeste et al., 2017). In mammalian cells, this family of channel proteins includes different members that are classified according to sequence similarity and solute permeability into: orthodox AQPs, aquaglyceroporins (GLPs) and superAQPs. The group of orthodox AQPs includes AQP0, AQP1, AQP2, AQP4, AQP5, AQP6, and AQP8, which are exclusively permeable to water. Aquaglyceroporins, which are permeable to water, glycerol, urea and other small electrolytes, include AQP3, AQP7, AQP9, and AQP10. Finally, the group of superAQPs comprises AQP11 and AQP12, which are localized in the membranes of intracellular organelles, present sequence variations to the other groups of AQPs and are involved in both water and glycerol transport.

Despite being ubiquitous, the distribution of AQPs across cell types and species is uneven and characteristic. Regarding mammalian sperm, AQP3, AQP7, and AQP11 have been identified in mouse (Yeung et al., 2009; Yeung and Cooper, 2010; Chen et al., 2011), human (Yeung et al., 2010; Laforenza et al., 2017), pig (Prieto-Martínez et al., 2014, 2015), cattle (Prieto-Martinez et al., 2016; Morató et al., 2018) and horse (Bonilla-Correal et al., 2017); AQP8 has been found in mouse (Yeung et al., 2009) and human spermatozoa (Yeung et al., 2010; Laforenza et al., 2017); and AQP9 has been reported to be present in pig sperm (Vicente-Carrillo et al., 2016). The main functions of AQPs in mammalian spermatozoa are volume regulation and osmoadaptation. In effect, AQP3 has a vital role in osmoregulation (Chen and Duan, 2011), which is of crucial importance in post-ejaculatory events, when sperm cells interact with the female tract. In addition, Prieto-Martínez et al. (2017) observed a positive correlation between relative AQP3-content and osmoadaptation during cryopreservation, which is a challenging process since sperm are exposed to significant osmolality changes. Moreover, AQP7 has been suggested to be related to sperm motility in humans (Saito et al., 2004), but not in mice (Sohara et al., 2007). As far as AQP9 is concerned, there is little evidence on its particular role, since despite having been observed in pig spermatozoa (Vicente-Carrillo et al., 2016), homozygous *Aqp9*^–^*^/^*^–^ mice show preserved fertility and their spermatozoa are morphologically normal and motile (Rojek et al., 2007). Finally, and with regard to AQP11, while its relative content was found to be correlated with plasma membrane integrity and motility in pig spermatozoa (Prieto-Martínez et al., 2014), it was confirmed as a cryotolerance biomarker in cattle (Morató et al., 2018) but not in pig spermatozoa (Prieto-Martínez et al., 2017). It is worth mentioning that the specific role of each AQP appears to differ across species, and that the particular relevance of their functions evokes that certain members of the AQP family can be compensated by others. Nevertheless, since exposure to the female reproductive tract involves a major challenge to the ability of spermatozoa to adapt to drastic osmolality variations, the role of AQPs in osmoregulation is essential to ensure preservation of sperm function and the success of subsequent post-ejaculatory events, such as motility activation [reviewed by Yeste et al. (2017)].

Because of their exposure to the female reproductive tract, sperm cells meet constantly changing environments, from which they receive different chemical and thermal signals. This set of inputs drives a series of signaling events that are essential for sperm capacitation, which is the process that confers sperm the ability to fertilize an oocyte (Chang, 1951). Briefly, after entering the female tract, spermatozoa find higher bicarbonate and calcium levels, whose influx activates the cAMP-PKA pathway and increases tyrosine phosphorylation downstream, thus hyperactivating sperm motility (reviewed by Puga Molina et al., 2018). In addition, this ion flow through plasma membrane triggers lipid reorganization and cholesterol efflux; as a consequence, the sperm membrane is hyperpolarized and its fluidity rises, which is crucial to increase membrane fusogenicity (reviewed in Bailey, 2010). Thereafter, progesterone triggers the acrosome reaction through its interaction with a G protein-coupled receptor (GPCR) (Buffone et al., 2014). Given the importance of intracellular pH as a regulator of the sperm-specific, soluble adenylyl cyclase (sAC), which is the source of cAMP that allows the activation of the cAMP-PKA pathway, it is reasonable to posit that AQPs might have a relevant role during mammalian sperm capacitation.

High affinity inhibitors are a reasonably accurate strategy to study AQPs function, and transitional metals have been used as blockers of water permeability in different cell types (Haddoub et al., 2009). Different mercurial compounds were used at first to prove the existence of water channels in erythrocytes and renal proximal tubule cells (Smith and Agre, 1991), as well as in *Xenopus* oocytes (Preston et al., 1992). Silver compounds have also been used as AQP inhibitors (Niemietz and Tyerman, 2002), showing a higher affinity and specificity for this family of proteins. Finally, cooper was also identified as a reversible, rapid and specific inhibitor of AQP3 (Zelenina et al., 2004).

Considering all the aforementioned, this study aimed to elucidate the role of the different AQPs in mammalian sperm capacitation using the pig as a model, and through their inhibition with transition metal compounds.

## Materials and Methods

### Animals and Ejaculates

A total of 32 ejaculates from separate Piétrain boars (*n* = 32) were used in this study. Boars were housed in a local farm (Grup Gepork SL, Masies de Roda, Spain) in controlled climatic conditions and fed with a standard diet. Sperm-rich fractions were manually collected and subsequently diluted to a final concentration of 33 × 10^6^ sperm/mL in a commercial semen extender (Vitasem LD; Magapor SL, Zaragoza, Spain) and preserved at 17°C. Samples were transported to the laboratory within 2 h post-collection and, upon arrival, ejaculates were combined in pools of four for each experiment. After that, each ejaculate pool was split into four different aliquots that were centrifuged (Universal 32R centrifuge; Hettich Zentrifugen; Tuttlingen, Germany) at 600 × *g* and 17°C for 5 min. Supernatants were discarded, and samples were then resuspended in pre-warmed, equilibrated capacitation medium [20 mM 4- (2-Hydroxyethyl) piperazine-1-ethanesulfonic acid (HEPES), 112 mM NaCl, 3.1 mM KCl, 5 mM glucose, 21.7 mM sodium L-lactate, 1 mM sodium pyruvate, 0.3 mM Na_2_HPO_4_, 0.4 mM MgSO_4_ × 7H_2_O, 4.5 mM CaCl_2_ × 2H_2_O, 5 mg/mL BSA and 15 mM bicarbonate; pH = 7.4 ± 0.1; osmolality = 0.300 Osm/Kg ± 0.01] to a final concentration of 25 × 10^6^ sperm/mL. Thereafter, the corresponding inhibitors were added according to the treatment, and samples were kept in a Hera Cell 150 incubator at 38°C and 5% CO_2_. In order to induce the acrosome reaction, progesterone (final concentration: 0.01 mg/mL) was added after 4 h of incubation and samples were incubated for an additional hour.

### AQP Inhibitors

Three different AQP inhibitors were added to capacitation medium prior to incubation: cooper sulfate (CuSO_4_; Sigma Aldrich; Saint Louis, MO, United States), mercury chloride (HgCl_2_; Sigma Aldrich) and silver sulfadiazine (AgSDZ; Sigma Aldrich). The final concentrations of each inhibitor were set after preliminary experiments based on previous studies (Niemietz and Tyerman, 2002; Yeung et al., 2009; Reca et al., 2018). Each inhibitor was previously diluted in water. CuSO_4_ (stock concentration: 400 mM) was added to samples at a final concentration of 100 μM; HgCl_2_ (stock concentration: 40 mM) was added to a final concentration of 20 μM; and AgSDZ (stock concentration: 40 mM) was used at a final concentration of 5 μM.

### Evaluation of Sperm Quality and Function

For the evaluation of sperm quality and function, sperm motility, flow cytometry, immunoblotting and ELISA analyses were performed after 0, 120, 240, 250, and 300 min of incubation in capacitation medium.

#### Motility

Sperm motility was assessed using a commercial computer-assisted sperm analysis (CASA) system, which consisted of a phase contrast microscope (Olympus BX41; Olympus, Tokyo, Japan) equipped with a video camera and ISAS software (Integrated Sperm Analysis System V1.0; Proiser SL, Valencia, Spain). With this purpose, a 5-μL drop of the sperm suspension was placed onto a Makler counting chamber (Sefi-Medical Instruments, Haifa, Israel) and observed under a negative phase-contrast field (Olympus 10 × 0.30 PLAN objective; Olympus). The following sperm motility parameters were recorded in each motility assessment: total motility (TMOT;%); progressive motility (PMOT,%); curvilinear velocity (VCL, μm/s); straight-line velocity (VSL, μm/s); average pathway velocity (VAP, μm/s); amplitude of lateral head displacement (ALH, μm); beat-cross frequency (BCF, Hz); linearity (LIN,%), that resulted from LIN = VSL/VCL × 100; straightness (STR,%), which was calculated as VSL/VAP × 100; and oscillation index (WOB,%), obtained from VAP/VCL × 100. A motile sperm was defined as having a VAP ≥ 10 μm/s, and a sperm cell was considered as progressively motile when its STR was at least 45%. In addition, the percentage of progressively motile spermatozoa among the population of motile sperm was calculated. For each sample, three replicates of at least 1,000 spermatozoa each were assessed. For each parameter, the corresponding mean and standard error of the mean (SEM) were calculated.

#### Flow Cytometry

Eight different parameters were assessed through flow cytometry in each time point and treatment: sperm plasma membrane integrity, membrane lipid disorder, acrosome membrane integrity, mitochondrial membrane potential (MMP), intracellular levels of calcium, superoxide (O_2_^–^•) radicals and hydrogen peroxide (H_2_O_2_), and intracellular pH. All fluorochromes were obtained from ThermoFisher Scientific (Waltham, MA, United States) unless otherwise stated. Prior to the addition of the corresponding combination of fluorochromes, samples were diluted in phosphate-buffered saline (PBS) to a final concentration of 2 × 10^6^ spermatozoa/mL. After staining, samples were incubated at 38°C in the dark. For each parameter, three replicates of at least 10,000 spermatozoa were assessed.

A Cell Lab Quanta^TM^ SC cytometer (Beckman Coulter; Fullerton; CA, United States) was used to analyze sperm samples. Samples were excited with an argon ion laser (488 nm) at a power of 22 mW. Cell diameter/volume was assessed using the Coulter principle, which measures electrical resistance changes caused by suspended, non-conductive particles in an electrolyte solution. In this system, forward scatter (FS) was replaced by electronic volume (EV) and for EV-channel calibration, 10-μm Flow-Check fluorospheres (Beckman Coulter) were positioned at channel 200 on the EV-scale.

For fluorescence detection, three different optical filters were used: FL1 (Dichroic/Splitter, DRLP: 550 nm, BP filter: 525 nm, detection width 505–545 nm); FL2 (DRLP: 600 nm, BP filter: 575 nm, detection width: 560–590 nm); and FL3 (LP filter: 670 nm/730 nm, detection width: 655–685 nm). FL1 was used to detect green fluorescence from SYBR-14, YO-PRO-1, fluorescein isothiocyanate (FITC)-conjugated peanut agglutinin (PNA; PNA-FITC), JC-1 monomers (JC-1_mon_), Fluo-3, 2′,7′-dichlorofluorescein (DCF^+^) and 2′,7′-Bis-(2-Carboxyethyl)-5-(and-6)-Carboxyfluorescein, Acetoxymethyl (BCECF). FL2 was used to detect orange fluorescence from JC-1 aggregates (JC-1_agg_). Finally, FL3 was used to detect red fluorescence from propidium iodide (PI), merocyanine 540 (M540) and ethidium (E^+^). The signal was logarithmically amplified, and photomultiplier settings were adjusted according to particular staining methods.

The sheath flow rate was set at 4.17 μL/min, and EV and side scatter (SS) were measured and linearly recorded for all particles. Subcellular debris (particle diameter < 7 μm) and cell aggregates (particle diameter > 12 μm) were excluded through the adjustment of the analyzer threshold on the EV channel, and the sperm-specific events were positively gated on the basis of EV/SS distributions. For each sample and parameter, three different replicates of a minimum of 10,000 events were evaluated.

Data analysis was performed using Flowing Software (Ver. 2.5.1; University of Turku, Finland), following the recommendations of the International Society for Advancement of Cytometry (ISAC). According to Petrunkina et al. (2010), the percentage of non-sperm debris particles from the SYBR-14^–^/PI^–^ population was used to correct the events corresponding to double-negative particles in the other protocols. For each sample and parameter, the corresponding mean and SEM were calculated.

##### Sperm plasma membrane integrity

The assessment of plasma membrane integrity was performed using the LIVE/DEAD sperm viability kit (Molecular Probes; Eugene, OR, United States) following the protocol of Garner and Johnson (1995). Briefly, spermatozoa were incubated in the presence of SYBR-14 (final concentration: 100 nmol/L) for 10 min, and PI (final concentration: 12 μmol/L) was subsequently added prior to an additional incubation of 5 min. Three different sperm populations were identified in flow cytometry dot-plots: (1) viable, green-stained spermatozoa (SYBR-14^+^/PI^–^); (2) non-viable, red-stained spermatozoa (SYBR-14^–^/PI^+^); and (3) non-viable, green- and red-stained spermatozoa (SYBR-14^+^/PI^+^). In addition, unstained, non-sperm particles (SYBR-14^–^/PI^–^) were not included for the calculation of the final percentages of the three previously mentioned populations. SYBR-14 fluorescence spill over into FL3 channel was compensated (2.45%).

##### Membrane lipid disorder

Co-staining with M540 and YO-PRO-1 was used to evaluate sperm membrane lipid disorder, following the protocol from Rathi et al. (2001) with minor modifications (Yeste et al., 2014). This protocol is based on the intercalation of M540 in the outer monolayer of the sperm plasma membrane when packing order of phospholipids decreases. In brief, samples were co-stained with M540 (final concentration: 2.6 μmol/L) and YO-PRO-1 (final concentration: 25 nmol/L) and then incubated for 10 min. Four different sperm populations were identified in flow cytometry dot-plots: (1) viable spermatozoa with low membrane lipid disorder (M540^–^/YO-PRO-1^–^); (2) viable sperm with high membrane lipid disorder (M540^+^/YO-PRO-1^–^); (3) non-viable sperm with low membrane lipid disorder (M540^–^/YO-PRO-1^+^); and (4) non-viable sperm with high membrane lipid disorder (M540^+^/YO-PRO-1^+^). The percentage of SYBR-14^–^/PI^–^ particles corresponding to non-sperm debris was used to correct the percentages of viable spermatozoa with low membrane lipid disorder (M540^–^/YO-PRO-1^–^); percentages of the other sperm populations were recalculated. Data were not compensated. Membrane lipid disorder was assessed through the calculation of the percentage of viable sperm with low membrane lipid disorder (M540^–^/YO-PRO-1^–^ sperm) from the population of viable spermatozoa (YO-PRO-1^–^ sperm).

##### Acrosome membrane integrity

Samples were co-stained with fluorescein isothiocyanate (FITC)-conjugated peanut agglutinin (PNA; PNA-FITC) and PI in order to evaluate acrosome membrane integrity following the protocol from Nagy et al. (2003). PNA-FITC binds specifically to the outer acrosomal membrane. In brief, samples were co-incubated in the presence of PNA-FITC (final concentration: 2.5 μg/mL) and PI (final concentration: 12 μmol/L) for 10 min. As spermatozoa were not permeabilized, four different sperm populations were identified in flow cytometry dot-plots: (1) spermatozoa with an intact plasma membrane (PNA-FITC^–^/PI^–^); (2) spermatozoa with a damaged plasma membrane and an outer acrosome membrane that could not be fully intact (PNA-FITC^+^/PI^+^); (3) spermatozoa with a damaged plasma membrane and a non-intact acrosome membrane (PNA-FITC^–^/PI^+^); and (4) spermatozoa a with damaged plasma membrane (PNA-FITC^+^/PI^–^). The percentage of SYBR-14^–^/PI^–^ particles corresponding to non-sperm debris was used to correct the percentages of viable sperm with intact acrosome (PNA-FITC^–^/PI^–^), and the percentages of the other sperm populations were recalculated. PNA-FITC fluorescence spill over into FL3 channel was compensated (2.45%).

##### Intracellular levels of calcium

Sperm samples were co-stained with Fluo-3 AM and PI for the evaluation of intracellular calcium levels following the protocol from Harrison et al. (1993). Fluo-3 stains calcium that is located in the mid-piece, but it also stains faintly the calcium present in the sperm head (Yeste et al., 2015). In brief, samples were co-incubated with Fluo-3 (final concentration: 1 μmol/L) and PI (final concentration: 12 μmol/L) for 10 min. Four different sperm populations were identified in flow cytometry dot-plots: (1) viable sperm with low intracellular levels of calcium (Fluo-3^–^/PI^–^); (2) viable sperm with high intracellular levels of calcium (Fluo-3^+^/PI^–^); (3) non-viable sperm with low intracellular levels of calcium (Fluo-3^–^/PI^+^); and (4) non-viable sperm with high intracellular levels of calcium (Fluo-3^+^/PI^+^). The percentage of SYBR-14^–^/PI^–^ particles corresponding to non-sperm debris was used to correct the percentages of viable sperm with low intracellular levels of calcium (Fluo-3^–^/PI^–^), and the percentages of the other sperm populations were recalculated. Fluo-3 fluorescence spill over into FL3 channel was compensated (2.45%). Intracellular levels of calcium were assessed through the calculation of the percentage of viable sperm with high intracellular levels of calcium (Fluo-3^+^/PI^–^ sperm) within the population of viable spermatozoa (PI^–^ sperm).

##### Mitochondrial membrane potential

Samples were stained with JC-1 in order to evaluate mitochondrial membrane potential following the protocol of Ortega-Ferrusola et al. (2008) with minor modifications. JC-1 molecules (5,5′,6,6′-tetrachloro-1,1′,3,3′tetraethyl-benzimidazolylcarbocyanine iodide) remain as monomers (JC-1_mon_) in the presence of low MMP and emit green fluorescence, whereas in the presence of high MMP, these molecules form aggregates (JC-1_agg_) that present orange fluorescence. Briefly, samples were incubated with JC-1 (final concentration: 0.3 μmol/L) for 30 min. Flow cytometry dot-plots allowed the identification of three different populations: (1) sperm with mitochondria presenting low MMP (JC-1_mon_; FL1^+^/FL2^–^); (2) sperm with mitochondria showing high MMP (JC-1_agg_; FL1^–^/FL2^+^); and (3) sperm with heterogeneous mitochondria (JC-1_mon_ and JC-1_agg_; FL1^+^/FL2^+^). The percentage of SYBR-14^–^/PI^–^ particles corresponding to non-sperm debris was used to correct the percentages of sperm with mitochondria presenting low MMP (JC-1_mon_; FL1^+^/FL2^–^), and the percentages of the other sperm populations were recalculated. The sum of populations (2) and (3) corresponded to high MMP-spermatozoa. MMP was also assessed through the calculation of the ratio of the intensity of fluorescence JC-1_agg_/JC-1_mon_ from each sperm population. JC1_mon_ fluorescence spill-over into the FL2 channel was compensated (51.70%).

##### Intracellular levels of superoxide (O_2_^–^•) radicals

Samples were co-stained with hydroethidine (HE) and YO-PRO-1 in order to evaluate the intracellular levels of superoxides (O_2_^–^•) following the protocol from Guthrie and Welch (2006). In the presence of O_2_^–^• radicals, HE is oxidized into ethidium (E^+^) and other products. Briefly, samples were incubated in the presence of HE (final concentration: 4 μmol/L) and YO-PRO-1 (final concentration: 25 nmol/L) for 20 min. As a result, flow cytometry dot-plots allowed the identification of four different sperm populations: (1) viable sperm with low intracellular levels of O_2_^–^• (E^–^/YO-PRO-1^–^); (2) viable sperm with high intracellular levels of O_2_^–^• (E^+^/YO-PRO-1^–^); (3) non-viable sperm with low intracellular levels of O_2_^–^• (E^–^/YO-PRO-1^+^); and (4) non-viable sperm with high intracellular levels of O_2_^–^• (E^+^/YO-PRO-1^+^). The percentage of SYBR-14^–^/PI^–^ particles corresponding to non-sperm debris was used to correct the percentages of viable sperm with low intracellular levels of O_2_^–^• (E^–^/YO-PRO-1^–^), and the percentages of the other sperm populations were recalculated. YO-PRO-1 spill-over into the FL3 channel was compensated (5.06%). Intracellular levels of superoxide radicals were assessed through the calculation of the percentage of viable sperm with high intracellular levels of superoxide (E^+^/YO-PRO-1^–^ sperm) within the population of viable spermatozoa (YO-PRO-1^–^sperm).

##### Intracellular levels of hydrogen peroxide (H_2_O_2_)

Intracellular levels of hydrogen peroxide (H_2_O_2_) were evaluated through co-staining with H_2_DCFDA and PI following the protocol from Guthrie and Welch (2006). In the presence of H_2_O_2_, the non-fluorescent probe H_2_DCFDA is intracellularly de-esterified and oxidized into highly fluorescent DCF^+^. In brief, samples were co-incubated with H_2_DCFDA (final concentration: 200 μmol/L) and PI (final concentration: 12 μmol/L) for 30 min. Four different sperm populations were identified in flow cytometry dot-plots: (1) viable sperm with low intracellular levels of H_2_O_2_ (DCF^–^/PI^–^); (2) viable sperm with high intracellular levels of H_2_O_2_ (DCF^+^/PI^–^); (3) non-viable sperm with low intracellular levels of H_2_O_2_ (DCF^–^/PI^+^); and (4) non-viable sperm with high intracellular levels of H_2_O_2_ (DCF^+^/PI^+^). The percentage of SYBR-14^–^/PI^–^ particles corresponding to non-sperm debris was used to correct the percentages of viable sperm with low intracellular levels of H_2_O_2_ (DCF^–^/PI^–^), and the percentages of the other sperm populations were recalculated. DCF^–^ spill-over into the FL3 channel was compensated (2.45%). Intracellular levels of hydrogen peroxide were assessed through the calculation of the percentage of viable sperm with high intracellular levels of peroxide (DCF^+^/PI^–^ sperm) within the population of viable spermatozoa (PI^–^ sperm).

##### Intracellular pH

Intracellular pH was measured through staining with BCECF following the protocol from Pons-Rejraji et al. (2009) with minor modifications. BCECF is intracellularly modified by esterases, and its fluorescence excitation profile is pH-dependent. In brief, samples were incubated in the presence of BCECF (final concentration: 6 μmol/L) for 25 min. Data were not compensated. The intensity of fluorescence was measured for the different treatments and time points, and the intracellular pH was calculated as the variation compared to the control.

#### Immunoblotting

Tyrosine phosphorylation levels were assessed for the different treatments and time points through immunoblotting. With this purpose, samples were centrifuged at 600 × *g* and capacitation medium was discarded. Samples were subsequently stored at −80°C until analysis.

For cell lysis, samples were thawed on ice and resuspended in lysis buffer (xTractor^®^ Buffer; Takara Bio, Mountain View, CA, United States) supplemented with 1% protease inhibitor cocktail, 1 mM sodium orthovanadate and 1 mM phenylmethanesulfonyl fluoride (PMSF). After incubation at 4°C for 30 min with shaking, samples were sonicated thrice for 30 s. Samples were then centrifuged at 4°C and 12,000 × *g* for 15 min, and supernatants were collected and stored at −80°C until analysis. Total protein was quantified by triplicate through a detergent compatible (DC) method (Bio-Rad, Hercules, CA, United States) using an Epoch^TM^ microplate spectrophotometer (BioTek, Winooski, VT, United States).

For each sample, 10 μg of total protein was diluted in loading buffer, which consisted of 4 × Laemmli reductor (Bio-Rad) supplemented with 10% (v:v) 2-Mercaptoethanol. Then, samples were incubated at 95°C for 5 min and a total volume of 30 μL per sample was loaded onto a gradient (8–16%) polyacrylamide gel (Mini-PROTEAN^®^ TGX Stain-Free^TM^ Precast Gels, Bio-Rad). Electrophoresis ran at 150 V for approximately 1 h, and proteins from the gels were transferred onto polyvinylidene difluoride (PVDF) membranes using *Trans-*Blot^®^ Turbo^TM^ (Bio-Rad). Next, total protein content was visualized by UV exposition and acquisition using a G:BOX Chemi XL system (SynGene, Frederick, MD, United States). Membranes were subsequently incubated in blocking buffer [10 mM Tris, 150 mM NaCl, 0.05% (v:v) Tween-20 and 5% (w:v) bovine serum albumin (Roche Diagnostics, S.L., Basel, Switzerland); pH = 7.3] in agitation for 1 h at room temperature. Then, membranes were incubated overnight at 4°C with a 4G10^®^ Platinum, Anti-Phosphotyrosine Antibody [mouse monoclonal cocktail IgG2b; Sigma Aldrich; 1:5,000; (v:v)]. Then, membranes were washed five times with TBS-Tween 20 1 × (10 mmol/L Tris, 150 mmol/L NaCl and 0.05% Tween-20; pH = 7.3) for 5 min before incubation with a rabbit anti-mouse, secondary antibody conjugated with HRP [ref. P0260; Agilent, Santa Clara, CA, United States; 1:10,000 (v:v)] for 1 h in agitation. Finally, membranes were washed five times. For visualization, membranes were incubated in a chemiluminescence substrate (Immobilion^TM^ Western Detection Reagents, Millipore) for 5 min, and then revealed in G:BOX Chemi XL 1.4.

Two technical replicates per sample were evaluated, and band quantification was performed using the Image Studio^TM^ Lite Software (LI-COR Biosciences GmbH, Bad Homburg vor der Höhe, Germany). Band pattern quantifications were normalized using total protein values, and the corresponding mean ± SEM was calculated.

#### Enzyme-Linked Immunosorbent Assays

Concentrations of PKA in sperm samples were measured using a colorimetric activity kit (EIAPKA; Invitrogen, ThermoFisher Scientific) following the manufacturer’s instructions. In brief, samples were centrifuged at 600 × *g*, capacitation medium was discarded and sperm cells were stored at −80°C until analysis. For cell lysis, samples were resuspended in cell lysis buffer, incubated for 30 min on ice with occasional vortexing, and then sonicated three times for 30 s each. After that, samples were centrifuged at 10,000 × *g* and 4°C for 10 min, and supernatants were recovered for analysis. Then, standards were diluted in kinase reaction buffer to obtain the standard curve (0.625, 1.25, 2.5, and 5 U/mL PKA), and samples were also diluted in this buffer (1:5,000). Then, 40 μL of the standards and diluted samples were added to the plate in duplicate, and 10 μL of ATP were also added to each well prior to a 90-min incubation at 30°C. After washing plate wells four times, 25 μL of the Donkey anti-Rabbit IgG HRP conjugate was added to each well followed by 25 μL of the Phospho PKA substrate antibody. Then, the plate was incubated at room temperature for 1 h. After washing the plate wells four times, 100 μL of TMB substrate was added to each well before incubating the plate at room temperature for 30 min. Finally, 50 μL of stop solution was added to all wells, and the plate was immediately read at 450 nm using an Epoch^TM^ microplate spectrophotometer (BioTek). Means were calculated for duplicate measurements of standards and samples, and average optical density from the blank was subtracted. The equation of the linear regression curve obtained through the interpolation of PKA concentration from absorbance reading was: (PKA) = 12.148 × Abs + 0.2793, *R*^2^ = 0.9975 for one plate, and (PKA) = 6.5865 × Abs—0.0454, *R*^2^ = 0.9962 for the other. This ELISA kit showed a sensitivity of 0.366 U/mL.

Concerning cAMP concentration, sperm samples were measured using a colorimetric competitive ELISA kit (ab133051; Abcam, Cambridge, United Kingdom) following the manufacturer’s instructions. Briefly, samples were centrifuged at 600 × *g* to eliminate capacitation medium and sperm cells were stored at −80°C until analysis. Lysis buffer (0.1 mol/L HCl and 1% Triton X-100) was used to resuspend sperm cells, which were incubated at room temperature for 30 min with agitation. Next, samples were sonicated three times for 30 s and cell debris were pelleted through centrifugation at 10,000 × *g* and 4°C for 10 min. Supernatants were recovered for analysis. Then, standards were diluted in lysis buffer to obtain the standard curve (0.078, 0.312, 1.25, 5, and 20 pmol/mL cAMP), and samples were also diluted in this buffer (1:20; v:v). Then, acetylating reagent [acetic anhydride diluted and triethylamine; 1:2 (v:v)] was added to standards and samples [1:20 (v:v)] to achieve the maximum sensitivity of the kit (0.039 nmol/L). Apart from standards and samples, different controls were also loaded onto the plate: blank wells, that only contained pNpp substrate; total activity wells, which contained conjugate and pNpp substrate; non-specific binding control, with neutralizing reagent, lysis buffer, conjugate and substrate; and the zero standard, that contained neutralizing reagent, lysis buffer, conjugate, antibody and substrate. Fifty μL of neutralizing reagent was added to the plate wells, prior to the addition of 100 μL of standards and samples. After that, 50 μL of Cyclic AMP complete Alkaline Phosphatase conjugate was added, followed by 50 μL of cyclic AMP complete antibody. Thereafter, the plate was incubated at room temperature for 2 h with shaking. Next, 200 μL of pNpp substrate solution was added to every well and the plate was incubated at room temperature for 1 h. Finally, 50 μL of stop solution was added to every well and the plate was then read at 405 nm using an Epoch^TM^ microplate spectrophotometer (BioTek). Means were calculated for duplicate measurements of standards and samples, and average optical densities from the blank and the non-specific binding control were subtracted. The equation of the exponential regression curve obtained through the interpolation of cAMP concentration from absorbance reading was: (cAMP) 11.16942 × e^–11.30138 × Abs^, *R*^2^ = 0.99001 for one plate, and (cAMP) = 29.322 × e^–24.69 × Abs^, *R*^2^ = 0.9821 for the other.

### Statistical Analyses

The results of this work were analyzed using a statistical package (IBM SPSS Statistics 25.0; Armonk, New York, United States). In order to assess the distribution of data and homogeneity of variances, Shapiro–Wilk and Levene tests were run, respectively. For each sperm parameter, a mixed model was subsequently conducted. The time point (i.e., 0, 120, 240, 250, or 300 min) was the intra-subjects factor, the treatment at a given concentration (i.e., control, CuSO, HgCl_2_ or AgSDZ) was the fixed-effects factor (inter-subject), and the ejaculate pool (i.e., 1–8) was the random-effects factor. Pair-wise comparisons were evaluated through a *post hoc* Sidak test, and the level of significance was set at *P* ≤ 0.05. Data are shown as mean ± standard error of the mean (SEM).

## Results

**FIGURE 1:**
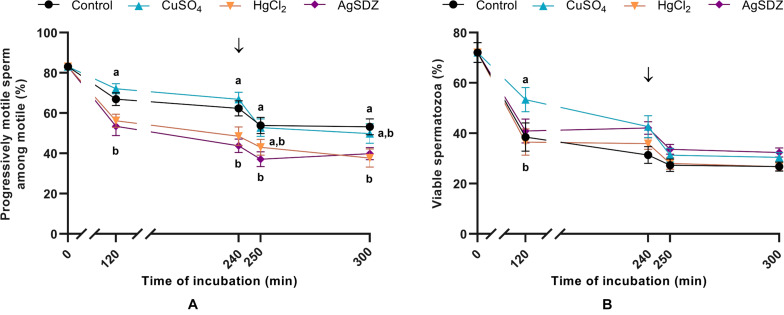
Sperm motility and viability. **(A)** Percentages of progressively motile sperm within the population of motile cells (%) and **(B)** sperm viability measured as the percentage of spermatozoa with an intact plasma membrane (%) in samples exposed to the presence or absence of different AQP inhibitors in the capacitation medium: cooper sulfate (CuSO_4_), mercury chloride (HgCl_2_) and silver sulfadiazine (AgSDZ). Data were collected after 0, 120 and 240 min of incubation in capacitation medium. At this point, progesterone was added to capacitation medium (arrow) and data were collected after further 10 min and 60 min of incubation. Data are shown as mean ± SEM, and different letters (a,b) indicate significant differences (*P* < 0.05) between different treatments within a given time point.

**FIGURE 2:**
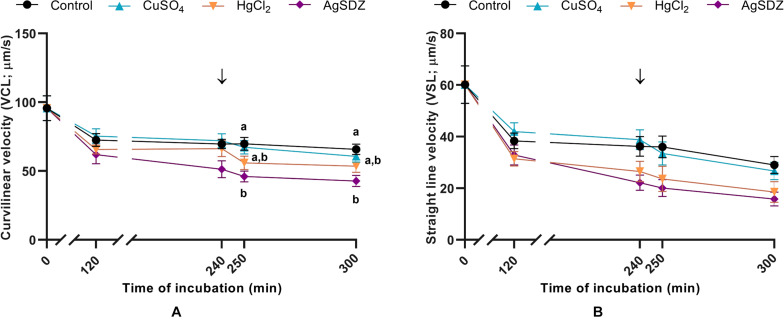
Sperm kinetics parameters. **(A)** Curvilinear velocity (VCL, μm/s) and **(B)** straight-line velocity (VSL, μm/s) in samples exposed to the presence or absence of different AQP inhibitors in the capacitation medium: cooper sulfate (CuSO_4_), mercury chloride (HgCl_2_) and silver sulfadiazine (AgSDZ). Data were collected after 0, 120 and 240 min of incubation in capacitation medium. At this point, progesterone was added to capacitation medium (arrow) and data were collected after further 10 min and 60 min of incubation. Data are shown as mean ± SEM, and different letters (a,b) indicate significant differences (*P* < 0.05) between different treatments within a given time point.

**FIGURE 3:**
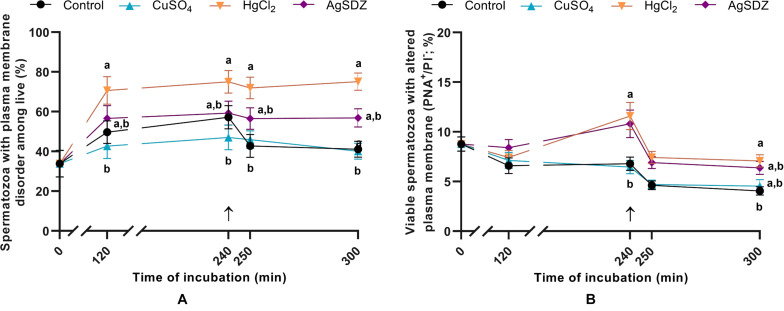
Sperm plasma and acrosome membranes status. **(A)** Percentages of sperm cells with high membrane lipid disorder within the viable sperm population (%) and **(B)** percentages of viable spermatozoa with altered integrity of acrosome membrane (%) in samples exposed to the presence or absence of different AQP inhibitors in the capacitation medium: cooper sulfate (CuSO_4_), mercury chloride (HgCl_2_) and silver sulfadiazine (AgSDZ). Data were collected after 0, 120 and 240 min of incubation in capacitation medium. At this point, progesterone was added to capacitation medium (arrow) and data were collected after further 10 min and 60 min of incubation. Data are shown as mean ± SEM, and different letters (a,b) indicate significant differences (*P* < 0.05) between different treatments within a given time point.

**FIGURE 4:**
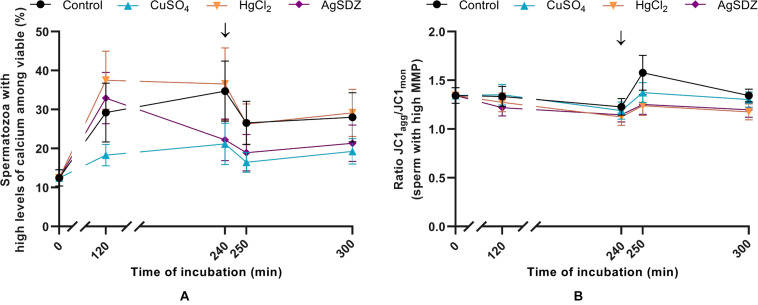
Intracellular levels of calcium and mitochondrial membrane potential. **(A)** Percentages of spermatozoa with high levels of intracellular calcium within the viable sperm population (%) and **(B)** ratio of JC1_agg_/JC1_mon_ intensity of fluorescence in the population of spermatozoa with high MMP in samples exposed to the presence or absence of different AQP inhibitors in the capacitation medium: cooper sulfate (CuSO_4_), mercury chloride (HgCl_2_) and silver sulfadiazine (AgSDZ). Data were collected after 0, 120 and 240 min of incubation in capacitation medium. At this point, progesterone was added to capacitation medium (arrow) and data were collected after further 10 min and 60 min of incubation. Data are shown as mean ± SEM, and different letters (a,b) indicate significant differences (*P* < 0.05) between different treatments within a given time point.

**FIGURE 5:**
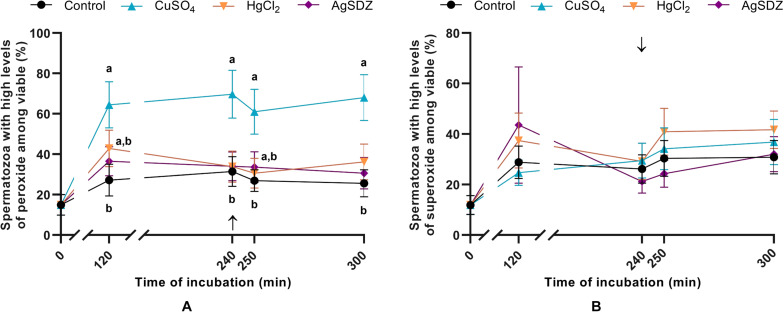
Intracellular levels of ROS. **(A)** Percentages of spermatozoa with high intracellular levels of hydrogen peroxide within the viable sperm population (%) and **(B)** percentages of spermatozoa with high intracellular levels of superoxide within the viable sperm population (%) in samples exposed to the presence or absence of different AQP inhibitors in the capacitation medium: cooper sulfate (CuSO_4_), mercury chloride (HgCl_2_) and silver sulfadiazine (AgSDZ). Data were collected after 0, 120 and 240 min of incubation in capacitation medium. At this point, progesterone was added to capacitation medium (arrow) and data were collected after further 10 min and 60 min of incubation. Data are shown as mean ± SEM, and different letters (a,b) indicate significant differences (*P* < 0.05) between different treatments within a given time point.

**FIGURE 6:**
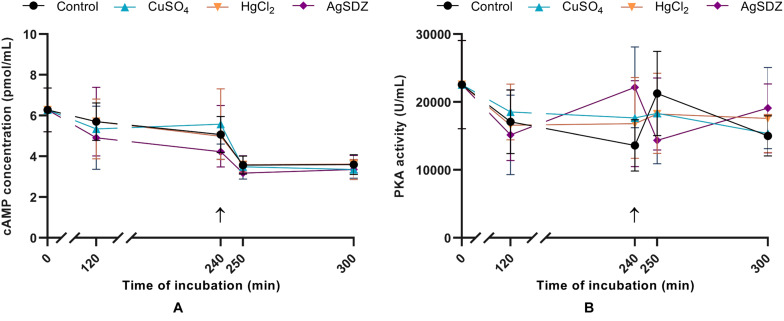
Intracellular concentration of cAMP and PKA activity. **(A)** Intracellular concentration of cyclic AMP (cAMP) in sperm cells (pmol/mL) and **(B)** PKA intracellular activity (U/mL) in samples exposed to the presence or absence of different AQP inhibitors in the capacitation medium: cooper sulfate (CuSO_4_), mercury chloride (HgCl_2_) and silver sulfadiazine (AgSDZ). Data were collected after 0, 120, and 240 min of incubation in capacitation medium. At this point, progesterone was added to capacitation medium (arrow) and data were collected after further 10 and 60 min of incubation. Data are shown as mean ± SEM, and different letters (a,b) indicate significant differences (*P* < 0.05) between different treatments within a given time point.

To determine the effects of AQP inhibitors during sperm capacitation, quality and function parameters of spermatozoa from the control and samples containing CuSO, HgCl_2_ or AgSDZ were evaluated after 0, 120, 240, 250 and 300 min of incubation. No differences between treatments and the control (*P* > 0.05) were expected at 0 min for any parameter measured, since inhibitors were added immediately before starting incubation in capacitation medium. Therefore, sperm quality was exclusively measured in the control samples at 0 min (Figures 1–6).

### Sperm Motility

Different motility and kinetics parameters were assessed in the presence of separate inhibitors and the control throughout incubation. From 120 min of incubation until the end of the period at 300 min, PMOT from the population of motile sperm (Figure 1A) was significantly lower in the treatments containing HgCl_2_ and AgSDZ than in the control (*P* < 0.05). Nevertheless, TMOT was significantly lower than the control after 120 min of incubation only (Table 1).

**TABLE 1:** Sperm motility and kinetics parameters from direct measurement.

**Time (min)**	**Control**	**CuSO_4_**	**HgCl_2_**	**AgSDZ**
	**TMOT (%)**			
0	62.71% ± 2.33	62.71% ± 2.33	62.71% ± 2.33	62.71% ± 2.33
120	35.60% ± 3.73^a^	40.13% ± 3.76^a^	22.09% ± 2.05^b^	19.29% ± 2.52^b^
240	30.87% ± 3.15	35.27% ± 3.77	21.01% ± 2.81	13.54% ± 2.47
250	16.24% ± 2.28	17.11% ± 2.70	10.00% ± 1.15	7.67% ± 1.04
300	16.11% ± 2.64	14.96% ± 2.45	8.09% ± 1.07	7.65% ± 1.28
	**VAP (μm/s)**			
0	69.87 ± 8.56	69.87 ± 8.56	69.87 ± 8.56	69.87 ± 8.56
120	46.98 ± 3.64	50.00 ± 4.16	40.23 ± 3.08	40.45 ± 4.66
240	44.76 ± 3.90	46.83 ± 4.27	37.06 ± 3.98	29.24 ± 3.60
250	45.35 ± 4.32	42.77 ± 4.82	32.10 ± 4.75	27.63 ± 3.40
300	38.27 ± 3.31	35.48 ± 3.67	27.49 ± 4.04	22.49 ± 2.83
	**ALH (μm)**			
0	3.05 ± 0.15	3.05 ± 0.15	3.05 ± 0.15	3.05 ± 0.15
120	2.57 ± 0.09	2.61 ± 0.11	2.40 ± 0.11	2.17 ± 0.20
240	2.42 ± 0.07^a^	2.51 ± 0.08^a^	2.42 ± 0.20^a,b^	1.84 ± 0.17^b^
250	2.64 ± 0.11^a^	2.41 ± 0.13^a^	1.87 ± 0.12^a,b^	1.53 ± 0.15^b^
300	2.39 ± 0.10^a^	2.04 ± 0.17^a,b^	1.75 ± 0.18^a,b^	1.46 ± 0.17^b^
	**BCF (Hz)**			
0	11.06 ± 0.64	11.06 ± 0.64	11.06 ± 0.64	11.06 ± 0.64
120	9.92 ± 0.56^a,b^	10.59 ± 0.47^a^	8.44 ± 0.45^a,b^	8.04 ± 0.74^b^
240	9.18 ± 0.55^a^	9.88 ± 0.52^a^	7.97 ± 0.88^a,b^	6.53 ± 0.89^b^
250	9.12 ± 0.74^a^	8.24 ± 0.63^a^	5.33 ± 0.63^b^	4.76 ± 0.63^b^
300	7.98 ± 0.55^a^	7.44 ± 0.74^a^	4.75 ± 0.72^b^	4.43 ± 0.68^b^

*Percentage of total motile spermatozoa (TMOT, %), average pathway velocity (VAP, μm/s), amplitude of lateral head displacement (ALH, μm) and beat-cross frequency (BCF, Hz) from samples exposed to the presence or absence of different AQP inhibitors in the capacitation medium: cooper sulfate (CuSO_4_), mercury chloride (HgCl_2_) and silver sulfadiazine (AgSDZ).*

*Data were collected after 0, 120, and 240 min of incubation in capacitation medium.*

*At this point, progesterone was added to capacitation medium (arrow) and data were collected after further 10 min and 60 min of incubation.*

*Data are shown as mean ± SEM, and different letters (a,b) indicate significant differences (*P* < 0.05) between different treatments within a given time point.*

Regarding kinetics parameters, VCL (Figure 2A) was significantly (*P* < 0.05) lower in the presence of AgSDZ than in control samples from 250 min of incubation in capacitation medium and until the end of the experiment. Similarly, samples containing AgSDZ presented lower ALH and BCF with respect to the control at 240, 250 and 300 min; whereas in the presence of HgCl_2_ samples presented the same effect from 250 min of incubation (*P* < 0.05; Table 1). In addition, STR was lower than the control in the presence of HgCl_2_ after 240 min and 300 min of incubation; in samples treated with AgSDZ, this effect was only observed after 300 min of incubation (*P* < 0.05; Table 2). Moreover, no significant differences between treatments/inhibitors and the control were observed in terms of VSL (Figure 2B), VAP, LIN, or WOB (*P* > 0.05) at any time point (Tables 1, 2).

**TABLE 2:** Secondary sperm kinetics parameters.

**Time (min)**	**Control**	**CuSO_4_**	**HgCl_2_**	**AgSDZ**
	**LIN (%)**			
0	61.18 ± 3.02	61.18 ± 3.02	61.18 ± 3.02	61.18 ± 3.02
120	52.56 ± 2.28	55.43 ± 2.03	47.52 ± 3.05	49.28 ± 4.66
240	50.57 ± 3.82^a,b^	53.45 ± 2.99^a^	38.71 ± 4.69^b^	41.22 ± 3.45^a,b^
250	50.51 ± 3.62	47.81 ± 3.74	39.88 ± 4.78	41.98 ± 3.56
300	43.05 ± 3.47	42.47 ± 3.85	31.48 ± 4.79	33.51 ± 3.59
	**STR (%)**			
0	86.31 ± 1.09	86.31 ± 1.09	86.31 ± 1.09	86.31 ± 1.09
120	81.41 ± 1.52	84.26 ± 1.44	77.23 ± 1.74	75.04 ± 5.94
240	79.07 ± 2.34^a^	82.30 ± 2.07^a^	66.13 ± 4.46^b^	71.72 ± 3.14^a,b^
250	77.57 ± 2.54	76.27 ± 2.28	68.24 ± 3.15	69.08 ± 2.45
300	73.41 ± 2.62^a^	72.58 ± 3.06^a,b^	59.68 ± 4.45^c^	62.38 ± 4.29^b,c^
	**WOB (%)**			
0	70.69 ± 3.24	70.69 ± 3.24	70.69 ± 3.24	70.69 ± 3.24
120	64.34 ± 2.35	65.64 ± 2.03	60.92 ± 2.80	60.40 ± 5.31
240	62.93 ± 3.33	64.35 ± 2.65	52.17 ± 4.37	55.97 ± 3.14
250	61.00 ± 3.61	61.78 ± 3.31	55.87 ± 4.12	59.42 ± 3.25
300	57.40 ± 2.96	56.90 ± 3.21	48.47 ± 4.01	49.42 ± 3.11

*Linearity (LIN, %), straightness (STR, %) and oscillation index (WOB, %) from samples exposed to the presence or absence of different AQP inhibitors in the capacitation medium: cooper sulfate (CuSO_4_), mercury chloride (HgCl_2_) and silver sulfadiazine (AgSDZ).*

*Data were collected after 0, 120, and 240 min of incubation in capacitation medium.*

*At this point, progesterone was added to capacitation medium (arrow) and data were collected after additional 10 and 60 min of incubation.*

*Data are shown as mean ± SEM, and different letters (a,b) indicate significant differences (*P* < 0.05) between different treatments within a given time point.*

### Sperm Plasma Membrane Integrity and Membrane Lipid Disorder

As aforementioned, sperm plasma membrane integrity was evaluated through co-staining with SYBR-14 and PI in order to assess the effects of separate inhibitors at the different time points (Figure 1B). Sperm viability was higher in the presence of CuSO_4_ than in the control after 120 min of incubation (*P* > 0.05).

Co-staining with M540 and YO-PRO-1 was used to evaluate the effects of the different inhibitors on plasma membrane lipid disorder during *in vitro* capacitation (Figure 3A). Percentages of spermatozoa exhibiting high levels of plasma membrane lipid disorder within the viable sperm population were significantly higher in samples containing HgCl_2_ than in the control after 250 and 300 min of incubation (*P* < 0.05). Nevertheless, a notable trend was observed from 120 min of incubation, since membrane lipid disorder was already higher in samples treated with HgCl_2_ than in the control (*P* = 0.062).

### Acrosome Membrane Integrity

Sperm were co-stained with PNA-FITC and PI to evaluate the effects of the different AQP inhibitors on acrosome membrane integrity during *in vitro* capacitation (Figure 3B). Samples treated with HgCl_2_ and AgSDZ presented a higher percentage of spermatozoa with an altered plasma/acrosomal membrane after 240 min of incubation (*P* < 0.05). At the end of the experiment (300 min), this difference persisted in samples treated with HgCl_2_ (*P* < 0.05).

### Intracellular Levels of Calcium

Intracellular levels of calcium were evaluated through co-staining with Fluo-3 AM and PI in order to assess the effects of different AQP inhibitors during capacitation (Figure 4A). There were no significant differences between the control and samples treated with inhibitors on the intracellular levels of calcium in the viable sperm population at any time point (*P* > 0.05).

### Mitochondrial Membrane Potential

Mitochondrial membrane potential (MMP) was assessed through staining with JC1 in order to evaluate the effect of different inhibitors on this sperm function parameter during capacitation (Figure 4B). In the population of sperm with high MMP, the ratio of JC1_agg_/JC1_mon_ intensity presented no significant differences between the different treatments and the control at any time point (*P* > 0.05).

### Intracellular Levels of ROS

The effects of different AQP inhibitors on the intracellular levels of ROS during capacitation were evaluated with two different co-staining protocols: H_2_DCFDA/PI was used to assess peroxide levels and HE/YO-PRO-1 was used to determine O_2_^–^• levels. The addition of CuSO_4_ to capacitation medium caused an increase in the percentage of viable spermatozoa with high intracellular levels of peroxides at any time point (*P* < 0.05; Figure 5A). No differences between the control and treated samples were observed in terms of O_2_^–^• levels at any time point (*P* > 0.05; Figure 5B).

### Intracellular cAMP Levels and PKA Activity

Two different Enzyme-Linked Immunosorbent Assays were conducted to assess PKA intracellular activity and intracellular cAMP levels. No significant differences in terms of cAMP concentration (Figure 6A) or PKA activity (Figure 6B) were observed between the control and AQP inhibitors at any time point.

### Intracellular pH

**FIGURE 7:**
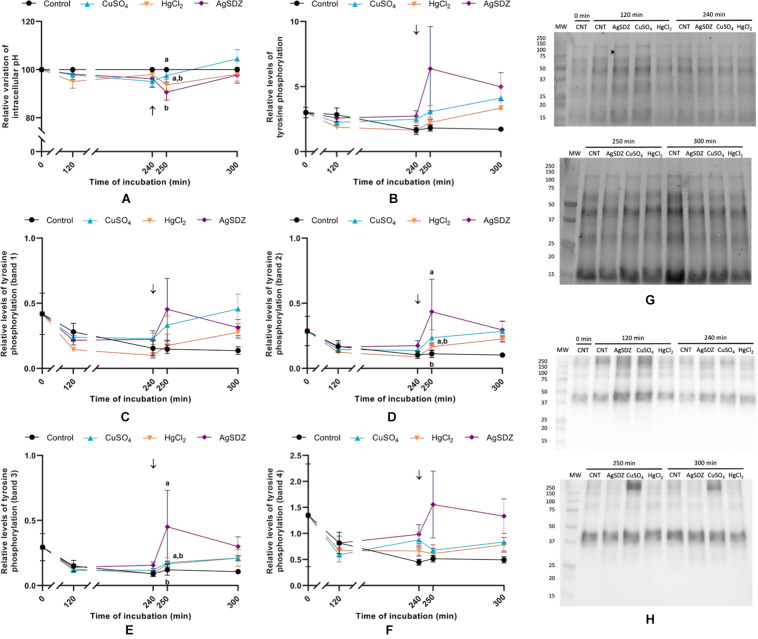
Relative variation of pH and relative levels of tyrosine phosphorylation. **(A)** Intracellular variation of pH compared to the control sample; **(B)** relative levels of total tyrosine phosphorylation; **(C)** relative levels of total tyrosine phosphorylation from band 1 (∼200 kDa); **(D)** relative levels of total tyrosine phosphorylation from band 2 (∼175 kDa); **(E)** relative levels of total tyrosine phosphorylation from band 3 (∼75 kDa); **(F)** relative levels of total tyrosine phosphorylation from band 4 (∼40 kDa); **(G)** total protein measured through stain-free; and **(H)** immunoblotting showing the tyrosine phosphorylation pattern after the incubation with a 4G10^®^ Platinum, mouse monoclonal Anti-Phosphotyrosine Antibody [1:5,000; (v:v)] and rabbit anti-mouse, secondary antibody conjugated with HRP [1:10,000; (v:v)]. All parameters were measured in samples exposed to the presence or absence of different AQP inhibitors in the capacitation medium: cooper sulfate (CuSO_4_), mercury chloride (HgCl_2_) and silver sulfadiazine (AgSDZ). Data were collected after 0, 120 and 240 min of incubation in capacitation medium. At this point, progesterone was added to capacitation medium (arrow) and data were collected after further 10 and 60 min of incubation. Data are shown as mean ± SEM, and different letters (a,b) indicate significant differences (*P* < 0.05) between different treatments within a given time point.

To assess the effects of inhibiting AQPs on intracellular pH, a BCECF staining protocol was used. The addition of AgSDZ caused a decrease in intracellular pH compared to the control after 250 min of incubation (*P* < 0.05; Figure 7A).

### Tyrosine Phosphorylation

Levels of tyrosine phosphorylation were assessed through immunoblotting. A tyrosine phosphorylation band pattern was observed, and apart from total phosphorylation, four different bands were analyzed to assess the potential differences between inhibitors and the control (Figures 7B–H). Despite no differences being observed in terms of total tyrosine phosphorylation of sperm proteins between different treatments and the control (*P* > 0.05), bands 2 and 3 from samples treated with AgSDZ presented a higher intensity than the control (*P* < 0.05), thus indicating higher levels of tyrosine phosphorylation.

## Discussion

Although several studies to address the function of ion channels during sperm capacitation were conducted (reviewed by Puga Molina et al., 2018), the potential role of water channels on this process is yet to be unveiled. Nevertheless, it is well known that soluble adenylate cyclase (sAC), which is regulated by pH, is crucial for the cAMP-PKA pathway triggered during capacitation (Chang and Oude-Elferink, 2014). Therefore, given the link between water flow and intracellular pH, it is reasonable to suggest that AQPs might be relevant in this process. For this reason, the aim of this study was to assess the role of the different groups of AQPs during mammalian sperm capacitation and acrosome reaction using the pig as a model. Three different salts of transition metals that have largely been demonstrated as AQP inhibitors (Haddoub et al., 2009) were used: cooper sulfate (CuSO_4_), mercury chloride (HgCl_2_) and silver sulfadiazine (AgSDZ). CuSO_4_ is a specific inhibitor of AQP3, since Cu^2+^ binds to three different extracellular residues of this protein, which correspond, in human AQP3, to Trp128, Ser152 and His241 (Zelenina et al., 2004). HgCl_2_ is an unspecific and reversible inhibitor of AQPs; its blocking effect presumably occurs because of the Hg^2+^-mediated, covalent modification of a Cys residue that is present in the pore of most AQPs. This modification either blocks the channel or causes conformational changes that avoid water transport (Hirano et al., 2010). Nevertheless, the fact that this Cys residue is not present in AQP7 could explain why previous studies demonstrated that AQP7 is Hg^2+^-resistant (Ishibashi et al., 1997). Finally, AgSDZ is an unspecific, irreversible inhibitor of all AQPs, and is also thought to interact with this Cys residue of the AQP pore. Such an irreversibility suggests that its inhibitory mechanism might be different from that of Hg^2+^. Niemietz and Tyerman (2002) surmised that, since the size of this ion (2.5 Å) matches the diameter of the AQP pore (2.8 Å), Ag^2+^ might block the AQP channel; thus, it is possible that this metal also inhibits AQP7 through that mechanism.

The effects of the aforementioned AQP inhibitors were evaluated in terms of sperm motility and kinetics parameters through a CASA system; flow cytometry was used to assess different sperm quality and function parameters; and tyrosine phosphorylation levels, PKA activity and intracellular levels of cAMP were evaluated as indicators of the intracellular pathways involved in capacitation. Briefly, the presence of CuSO_4_ led to higher sperm viability than the control and increased intracellular levels of peroxides after 120 min of incubation; this effect persisted even after the addition of progesterone to trigger the acrosome reaction at 240 min. The addition of HgCl_2_ augmented membrane lipid disorder from 120 min until the end of the experiment, caused a rise in the percentage of spermatozoa with an altered acrosome membrane from 240 min to the end of the experiment, and decreased progressive motility and other kinetics parameters from 120 min of incubation. Finally, the addition of AgSDZ caused a decrease in different parameters that evaluated sperm motility and kinetics during the entire incubation period, and the same effects on acrosome membrane integrity that were observed in the presence of HgCl_2_. In addition, the presence of AgSDZ caused a decrease in intracellular pH, as well as an increase in tyrosine phosphorylation levels after the addition of progesterone (250 min of incubation).

Regarding the effects of CuSO_4_, it became apparent from our results that AQP3 has not a key role in sperm capacitation or acrosome reaction. On the one hand, the higher percentages of sperm viability compared to the control after 120 min of incubation could be misleading. Considering the capacitation process leads to plasma membrane destabilization, it is possible that PI enters viable, membrane-destabilized sperm. Thus, while a population of sperm stained with both SYBR-14 and PI was observed, it could not be considered as strictly viable, since their viability was already compromised. In addition, the presence of CuSO_4_ led to a lower (although not significant) membrane lipid disorder compared to the control. This could explain the higher rate of entrance of PI into live cells in the control compared to sperm treated with CuSO_4_. These slight differences could lead to the small (but significant) difference in sperm viability between the control and the CuSO_4_ treatment. On the other hand, the increase in intracellular levels of peroxides could be explained because of AQP3 permeability to this molecule (Miller et al., 2010). Therefore, AQP3-inhibition could block the outflow of peroxides to the extracellular medium, which would make them to accumulate intracellularly. In fact, Delgado-Bermúdez et al. (2019) also reported an increase of intracellular peroxides in pig spermatozoa in the presence of 1,3-propanediol, another inhibitor of AQPs. Surprisingly, and despite high extracellular peroxide levels having a detrimental effect upon sperm function (Laforenza et al., 2017), no sperm quality or function parameter was altered when peroxides were intracellularly accumulated. This suggests that sperm capacitation and acrosome reaction, at least in pigs, do not seem to be impaired by high intracellular H_2_O_2_ levels. Nevertheless, ROS have a central role as stimulators of capacitation that has been extensively described in other mammalian species (reviewed by Aitken, 2017). In fact, Rivlin et al. (2004) suggested that while relatively low concentrations of H_2_O_2_ are beneficial for sperm capacitation, too high ones inhibit that process. This report also concluded that H_2_O_2_ activates adenylyl cyclase to produce cAMP, leading to PKA-dependent protein tyrosine phosphorylation. In addition, H_2_O_2_ has been demonstrated to inhibit different tyrosine phosphatases (Hecht and Zick, 1992), and Aitken (2017) suggested that in sperm this inhibition could increase phosphorylation of cAMP-PKA pathway target proteins. In spite of this, in this work, high intracellular H_2_O_2_ levels did not seem to stimulate capacitation-associated changes, either in terms of acrosome membrane integrity or in terms of cAMP levels, PKA activity or tyrosine phosphorylation patterns. Therefore, it seems that the observed increase in intracellular peroxides was not sufficient either to impair or to enhance capacitation or acrosome reaction. Additional studies are needed to evaluate the relevance of H_2_O_2_ in pig sperm capacitation.

Concerning HgCl_2_, it inhibits the different AQPs present in mammalian sperm except AQP7 (i.e., AQP3, AQP8, AQP9, and AQP11). Samples treated with HgCl_2_ showed an increase in membrane lipid disorder and impaired kinetics parameters, which could result from cholesterol efflux and lipid reorganization, since this is a featured event that occurs during capacitation (reviewed by Aitken, 2017). In this context, one could posit that certain capacitation-associated events are enhanced when some AQPs are inhibited. Nevertheless, the absence of significant effects on acrosome membrane integrity and on intracellular calcium levels after the addition of progesterone suggests that plasma membrane alterations are not related to a higher sperm ability to elicit *in vitro* capacitation and the subsequent acrosome reaction. This is supported by the fact that neither tyrosine phosphorylation, nor cAMP levels or PKA activity were altered. Thus, while the presence of HgCl_2_ seemed to induce an increase in membrane lipid disorder, likely increasing membrane fusogenicity, the observed changes were capacitation-like events that did not drive a proper, physiological sperm capacitation, as they did not lead to an increase in the percentage of spermatozoa undergoing the acrosome reaction. Considering all the aforementioned, these results suggest that AQP3 and AQP11 might exert some influence on certain capacitation-associated changes, but do not have direct consequences on the ability of spermatozoa to undergo the acrosome reaction. A possible reason for the observed effects would be that the limitation of water flow through the plasma membrane resulting from AQPs blocking would compromise sperm osmoregulation ability, which is in turn crucial for the activation and maintenance of sperm motility (reviewed by Chen and Duan, 2011). In our experiment, blocking of AQPs during *in vitro* capacitation might have caused an alteration in sperm volume, which could have ended up with plasma membrane damage and motility impairment, without altering the intracellular pathways involved in sperm capacitation.

Finally, AgSDZ, an unspecific inhibitor of all AQPs had similar effects to those observed in the presence of HgCl_2_. Nevertheless, an additional effect was observed, which suggests a relevant role of all AQPs as a whole during sperm capacitation and acrosome reaction. Samples treated with AgSDZ showed a lower intracellular pH compared to the control after the addition of progesterone to trigger the acrosome reaction, with a concomitant increase in tyrosine phosphorylation at the same time point (250 min of incubation). Nonetheless, cAMP levels and PKA activity were not impaired in the presence of this inhibitor. Therefore, either the concentration and activity changes were too subtle to be detected through the methods used in this study, or other signaling pathways were involved in the observed alteration of tyrosine phosphorylation patterns. According to Buffone et al. (2014), capacitation and acrosome reaction are driven through different signaling pathways. On the one hand, capacitation-associated changes are driven by phosphorylation of tyrosine residues as a downstream effect of the cAMP-PKA pathway, which relies upon cAMP synthesis by sAC. On the other hand, acrosome reaction is triggered by progesterone, which interacts with a GPCR coupled to a transmembrane adenylate cyclase (trAC) that is the source of the cAMP required in this process. It is important to highlight that, whilst sAC is regulated by pH, trAC activity does not. As the change in pH caused by the blockade of AQPs occurred after the addition of progesterone, it would be reasonable to suggest that sAC activity was unaltered, which would be in agreement with the lack of variations in PKA activity and cAMP levels. A potential candidate to explain the observed alteration in tyrosine phosphorylation levels would be PKC, which is involved in the intracellular signaling pathways that trigger the acrosome reaction (Teijeiro et al., 2017). Therefore, changes in osmoregulation during capacitation could induce latent alterations in sperm function that would become apparent upon triggering the acrosome reaction. The fact that these effects are only observed when all AQPs are inhibited as a whole might be the consequence of a functional compensation that is only possible when some AQPs remain unblocked. When a general inhibitor like AgSDZ is added and the function of all AQPs is suppressed, the absence of osmoregulation becomes an unbeatable challenge for the sperm cell that ends up altering its physiology, including the intracellular signaling pathways involved in the acrosome reaction. Additional experiments evaluating the mechanisms through which AQPs inhibition ends up with changes in intracellular pH and tyrosine phosphorylation patterns after triggering the acrosome reaction through progesterone are needed.

In conclusion, the relevance of AQPs during sperm capacitation is apparently related to their osmoregulatory ability. Whereas AQP3 is essential for H_2_O_2_ efflux, its inhibition does not seem to cause a toxic accumulation of this molecule at an intracellular level. Finally, the AQPs as a whole seem to have a relevant role for the intracellular signaling pathways involved in the acrosome reaction, thus suggesting that their blockade during the capacitation process has underlying effects that only become evident after the addition of progesterone.

## Data Availability Statement

The original contributions presented in the study are included in the article/supplementary material, further inquiries can be directed to the corresponding author/s.

## Author Contributions

AD-B and MY: conceptualization. AD-B, SR, and MY: methodology. AD-B, AS, and MY: formal analysis. AD-B, SR, ML, YM-O, AS, IB, and JR-M: investigation. MY: supervision and funding acquisition. AD-B: writing—original draft preparation and visualization. SR, ML, YM-O, AS, IB, JR-M, and MY: writing—review and editing. All authors have read and agreed to the published version of the manuscript.

## Conflict of Interest

The authors declare that the research was conducted in the absence of any commercial or financial relationships that could be construed as a potential conflict of interest.

## Publisher’s Note

All claims expressed in this article are solely those of the authors and do not necessarily represent those of their affiliated organizations, or those of the publisher, the editors and the reviewers. Any product that may be evaluated in this article, or claim that may be made by its manufacturer, is not guaranteed or endorsed by the publisher.
